# Genetic Susceptibility Is One of the Determinants for Severe Fever with Thrombocytopenia Syndrome Virus Infection and Fatal Outcome: An Epidemiological Investigation

**DOI:** 10.1371/journal.pone.0132968

**Published:** 2015-07-24

**Authors:** Jimin Sun, Yuming Tang, Feng Ling, Yue Chang, Xiaohong Ye, Wen Shi, Lei Zhang, Zhiping Chen, Haijiang Lin, Zaiping Qiu, Yanjun Zhang, Rong Zhang, Haiyan Mao, Enfu Chen, Junfen Lin, Jianmin Jiang, Shichang Xia, Zhenyu Gong

**Affiliations:** 1 Zhejiang Provincial Center for Disease Control and Prevention, Hangzhou, China; 2 Linhai Center for Disease Control and Prevention, Linhai, China; 3 Taizhou Municipal Center for Disease Control and Prevention, Taizhou, China; University of Texas Medical Branch, UNITED STATES

## Abstract

Severe fever with thrombocytopenia syndrome (SFTS) is an emerging infectious disease in China and case-fatality rate of SFTS is very high (approximately 10%). However, genetic susceptibility for SFTS virus (SFTSV) infection and fatal outcome of SFTSV infection in humans are unclear. In this study, we investigated the clinical, laboratory and epidemiological features of SFTS in a cluster of three sisters who died of SFTSV infection between late April and mid-May 2014. Before disease onset, two of the sisters (Case A and case B) had common exposure history for ticks by working together in a field to pick tea leaves from April 8 to April 12. The third sister (Case C) did not live or work together with case A and B, but had ticks in her living environment. SFTSV RNA sequences were amplified from three cases were not identical, suggesting that the three sisters were most likely infected with SFTSV through tick bite rather than through person-to-person transmission of SFTSV. The sequence of SFTSV from case C was identical to SFTSV sequences from 3 groups of ticks collected around the residential area of case C. Seroprevalence of SFTSV IgG antibody among healthy population in the area where the patients resided was 4.05% (3/74). The majority of SFTSV infections were mild cases and all three sisters died of SFTSV infection suggested that they were highly susceptible to SFTSV. Our findings indicated that genetic susceptibility was a risk factor for SFTSV infection and fatal outcome.

## Introduction

Severe fever with thrombocytopenia syndrome (SFTS) is an emerging hemorrhagic fever that was reported recently in rural areas of China. The causative agent of SFTS is a novel bunyavirus named SFTS virus (SFTSV), a novel *Phlebovirus* in the *Bunyaviridae* family. The disease is characterized by fever, thrombocytopenia and leukopenia, with a reported case-fatality rate ranging from 2.5% to 30% in different areas of endemicity [1–3].

The risk factors for fatal outcome of SFTS infection have been studied in China. Liu W et al reported that older age, decreased level of consciousness, and elevated levels of lactate dehydrogenase and creatine kinase were significantly associated with fatal outcome of SFTSV infection [4]. Fatal outcome of SFTSV was also believed to be associated with high viral RNA load in blood at admission, higher serum liver transaminase levels, more pronounced coagulation disturbances, and higher levels of acute phase proteins, cytokines, and chemokines.^3^ Similarity, Gai ZT et al reported that a period of 7–13 days after the onset of illness was the critical stage and the key risk factors that contributed to patient death were elevated serum aspartate aminotransferase, lactate dehydrogenase, creatine kinase, creatine kinase fraction, the appearance of CNS (central nervous system) symptoms, hemorrhagic manifestation, disseminated intravascular coagulation, and multi-organ failure [5]. Sun YL et al confirmed that Cytokines IL-1RA, IL-6, IL-10, G-CSF, IP-10, and MCP-1 were elevated in SFTS patients and produced at robust levels in fatal cases [6]. Between late April and mid-May 2014, a cluster of SFTSV infection occurred in southeastern China and three sisters contracted SFTSV infection and all of them deceased. This cluster informed us that death of SFTS patient may be probably related to genetic susceptibility.

## Materials & Methods

### Case Definition and Sample Collection

According to “the diagnosis and treatment programs of severe fever with thrombocytopenia syndrome” issued by The Ministry of Health of China [7], a suspected SFTS patient is defined as having the following clinical signs and symptoms: acute onset of fever (≥38.0°C) and other symptoms (e.g. gastrointestinal symptoms, bleeding), epidemiological risk factors (being a farmer or being exposed to ticks two weeks before onset of illness) and laboratory data consisting of thrombocytopenia and leukopenia was defined as a suspected case. A laboratory confirmed cases of SFTSV infection were defined as those who met the criteria for having a suspected case of SFTS and detection of SFTSV RNA by a molecular method or isolation of SFTSV from the patient’ blood or tissues.

Serum samples of suspected patients were collected within two weeks after onset of the illness and serum samples of family members, hospital staff who provided medical care for any of the patients and healthy persons living in the same areas with the patients were also collected. Moreover, ticks and serum samples of domesticated animals in the villages where patients lived were also collected.

### Laboratory Investigation

RNA was extracted from samples of suspected patients, ticks and domesticated animals using a high pure viral RNA kit according to the manufacturer’s instructions. SFTSV M genomic segments were amplified using specific primers and probes by RT-PCR assay as described previously [8]. PCR products were sequenced on both strands by Shanghai Sangon Biotechnology Co. (Shanghai, China).

Serum samples of hospital staff, patients’ family members, and healthy persons were tested for the presence of SFTSV specific IgG antibodies using an ELISA kit as described previously [9].

### Epidemiological Investigation

The aims of our study were explained to all patients and their consent was obtained prior to inclusion in this study. A standardized questionnaire was used to collect information about demographics, exposure history, clinical signs and symptoms, date of illness onset, date of first medical visit, date of hospitalization, date of specimen collection, and date of confirmation.

### Phylogenetic Analyses

Our sequences were compared to published sequences using the BLAST program from the National Center for Biotechnology Information Website (http://www.ncbi.nlm.nih.gov/BLAST/), and phylogenic analysis was performed using MEGA 4.0. For each gene analyzed, a phylogram was constructed by the neighbor-joining method. Confidence values for individual branches of the resulting tree were determined by bootstrap analysis with 1000 replicates.

### Ethics Statement

Experimental research reported in this study has been performed with the approval of the ethics committee of Zhejiang Provincial Centre for Disease Control and Prevention (Zhejiang CDC). Human research was carried out in compliance with the Helsinki Declaration. All adult subjects provided informed consent, and a parent or guardian of any child participant provided informed consent on their behalf, and informed consent given was written. The individuals' next-of kin have given written informed consent to publish these case details.

## Results

### Patients

**Fig 1:**
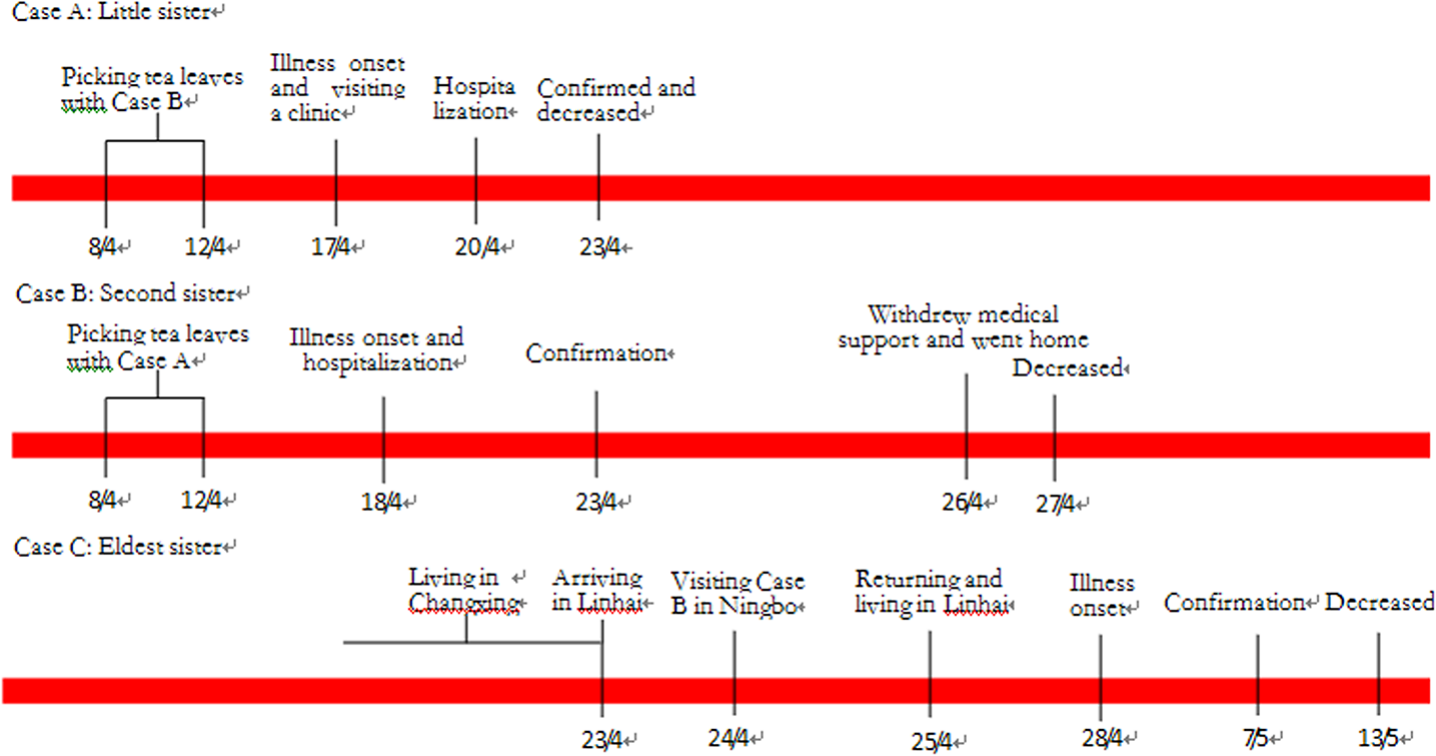
Timelines of key events for three SFTSV infections in southeastern China.

The cluster of SFTSV infection occurred in Linhai County, Zhejiang Province which locates in southeastern China. On April 17, 2014, a 62-year-old woman (case A) had sudden onset of fever (38.5°C), fatigue, myalgia, headache, anorexia, and abdominal pain and she was treated with cefradine and archie enzyme at the town hospital (Table 1). On April 19, she visited a county hospital in Linhai County and then she was admitted to a municipal hospital on April 20. On April 22, she developed high fever (39.2°C), obnubilation and septic shock and laboratory testing performed revealed leukopenia (WBC count, 1.0×10^9^/L) and thrombocytopenia (PLT count, 39×10^9^/L). The next day, she died of pulmonary hemorrhage and multi-organ failure (Fig 1). Serum sample of the patient was collected in the morning of April 23 and was confirmed to positive for SFTSV in the evening.

**Table 1:** The Clinical features of patients involved in a family cluster occurred in 2014, Southeastern China.

	Case A	Case B	Case C
General information			
Age, years and gender	62, female	65, female	79, female
Occupation	Farmer	Farmer	Farmer
Relationship with Case A	NA	Second sister	Eldest sister
Clinical manifestations			
Date of onset	April 17	April 18	April 28
Date of first medical visit	April 17	April 18	April 28
Date of hospitalization	April 20	April 18	May 5
Date of specimen collection	April 23	April 23	May 6
Date of confirmation	April 23	April 23	May 7
Date of death	April 23	April 27	May 13
Max temperature (°C)*	39.2	39.2	39.1
Symptoms	Fatigue, myalgia, headache, anorexia, abdominal pain	Fatigue, conjunctival congestion, nausea, vomiting, abdominal pain, diarrhea	Fatigue, anorexia, nausea, vomiting, lymphadenopathy
Blood counts			
White-cell count (109/L)*	1	0.8	1.4
Platelets count (109/L)*	39	25	24
Underlying diseases	NA	NA	Hypertension, heart diseases

*Data are peak or nadir measurement during hospitalization

Case B, the second sister of case A, was a female farmer aged 65. She had onset of fever, fatigue, conjunctival congestion, nausea, vomiting, abdominal pain, and diarrhea on April 18. She was admitted to a municipal hospital in Ningbo City which is adjacent to Linhai County on the same day. As the illness became worse, family members requested to withdraw intensive medical support, taking her home on April 26, where she deceased the next day (Fig 1).

Case C, the eldest sister of case A and case B, had onset of fatigue on April 28. She visited a town hospital from April 28 to May 3. On May 5, she was admitted to a local county hospital because of fever (39.1°C), fatigue, anorexia, nausea, and vomiting. In the hospital, she was treated with ribavirin and levofloxacin.

She deceased on May 13 because of multi-organ failure (Fig 1). Of note, she had underlying diseases including hypertension and heart diseases whereas case A and case B didn’t have underlying diseases.

### Epidemiological Investigations

Case A and case B picked tea leaves from April 8 to April 12 in a hilly area around the village where case B used to live in. Then case A return to her home in another town and case B visited her daughter in Ningbo City on April 13 (Fig 1). Ecological survey revealed no obvious change in mosquito density nor rising morbidity or mortality of domesticated animals at that time was observed in the village. But ticks were found in the tea garden where the sisters worked and body surfaces of cattle in the village and 92 *Haemaphysalis longicornis* adult ticks were collected in this area.

Case C used to live with her daughter’s family in Changxing County which locates in northwestern Zhejiang Province before April 23. She went to Linhai County on April 23 and lived there that night. Then she went to Ningbo City and visited case B on April 24. Because she was afraid to contract the disease from case B, she didn’t closely contact with case B and leaved the ward not more than 10 minutes. On April 25, she went back to her hometown in Linhai County, another village belong to the same town of case B’s hometown and lived at her home until May 5 (Fig 1). 88 *Haemaphysalis longicornis* nymph ticks were collected around the house where she lived in Linhai.

### Laboratory Investigations

**Fig 2:**
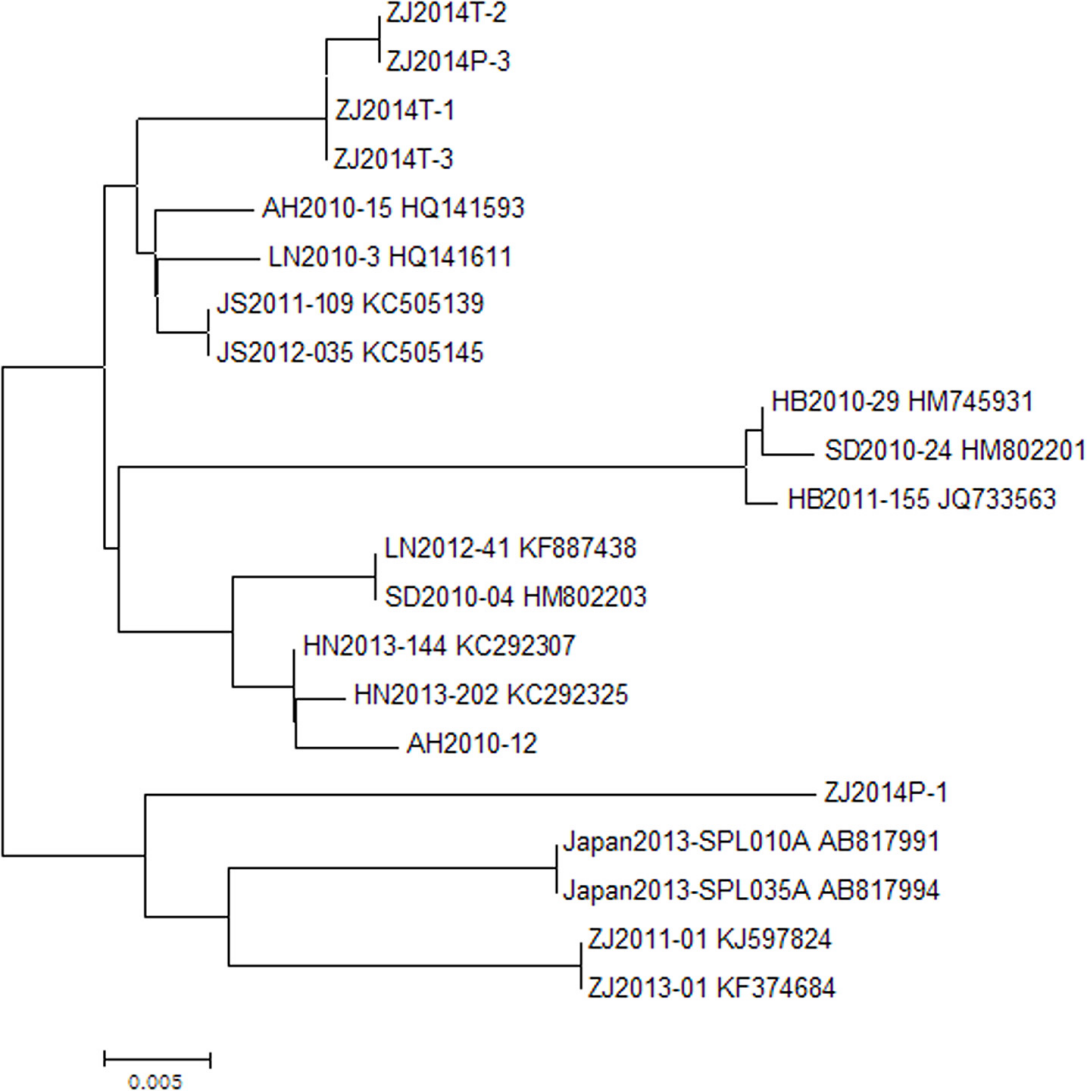
Phylogenetic analyses of SFTSV M genomic segments identified in patients and ticks.

SFTSV M genomic segments were detected in three groups of ticks which comprised 45 nymph ticks around the house of case C. No SFTSV M genomic segments were found in ticks from the tea garden and body surfaces of cattle in the village of case B, serum samples of 7 chickens, 6 dogs, 5 cows and 3 ducks from case C’ home. As shown in Fig 2, SFTSV M genomic segment of case C (ZJ2014P-3) were closely related to corresponding segments from the three tick groups (ZJ2014T-1, ZJ2014T-2, ZJ2014T-3) and corresponding segments from Jiangsu Province, Shandong Province, Henan Province, and Liaoning Province. SFTSV M genomic segment of case A (ZJ2014P-1) was most similar to corresponding segments from Japan and Zhoushan City which lies in the northeast of Zhejiang Province. But sequences of case A, case C, and corresponding sequences from other provinces were possessed high degrees of similarity between nucleotide (95%-99%).

SFTSV specific IgG antibodies were found in 2 of 47 serum samples from healthy persons living in the same areas and 1 (the son of case C) of 16 serum samples from family members. No SFTSV specific IgG antibodies were found in 11 serum samples from hospital staff who provided medical service for the patients.

## Discussion

Here, we reported a cluster of SFTSV infection in southeastern China. Three sisters contracted the disease and all of them deceased. Investigation studies revealed that they were infected with SFTSV through tick bites and no person-to-person transmission occurred. Firstly, case A and case B had obvious exposure history of picking tea leaves from April 8 to April 12 and ticks were collected around the tea garden. Secondly, Case A and case B developed the disease 5–10 days after the exposure which was compatible with the incubation period of SFTSV infection. Thirdly, although case C had history of visiting case B, she didn’t closely contact with her and the visit was brief. Fourthly, ticks were collected around the house where case C lived and SFTSV specific segments were found in these ticks. Finally, phylogenetic analyses revealed that SFTSV M genomic segment of case C was identical to corresponding segments from ticks collected around her residential place and sequences of case A and case C were not identical.

Of interesting, these three sisters lived in different cities before the cluster occurred. Case A lived in Linhai County, case B lived with her daughter’s family in Ningbo City, and Case C lived in Changxing County. But they were all infected with SFTSV through tick bites and all deceased. Case B only lived in Linhai County for five days and case C only lived in Linhai County for four days before the illness onset. These findings suggested that genetic susceptibility was one of determinants for SFTSV infection and fatal outcome. Firstly, all three sisters contracted the disease and deceased, but seroprevalence of SFTSV IgG indicated that only 4.05% (3/74) of samples from individuals living in the same areas were seropositive. Moreover, the SFTSV infection of individuals whose samples were seropositive was asymptomatic. Secondly, there was no obvious mutation in SFTSV detected from case A and case C although they were not identical. Sequences of case A, case C, and corresponding sequences from other provinces were possessed high degrees of similarity between nucleotide (95%-99%). These findings indicated that the infection and fatal outcome of three sisters were not related to virus mutation. Thirdly, many clusters of SFTSV infection were reported in China and these studies showed that SFTSV infection of family members of patient was very common [10–16]. Furthermore, another study reported that seroprevalence of SFTSV among people who were family members of patient was significantly high. Fourthly, some viruses have been confirmed to be more easily transmitted between individuals with genetic connection [17]. For example, genetic susceptibility is one of determinates for Hantaan virus, Dengue virus, West Nile virus, H5N1, and H1N1 infection [18–23]. Finally, underlying diseases might be risk factors for SFSTV infection and fatal outcome. But case A and case B didn’t have underlying diseases. We could rule out the influence of underlying diseases for the infection and death of case A and case B.

There were several limitations to our study. Firstly, we could not isolate virus from patients and ticks. Secondly, we could not get viral sequence of case B. Therefore, we didn’t know the similarity of corresponding sequences of case A and case B. Thirdly, all three patients deceased and samples of them were very limited which prevent further study on genetic susceptibility of the disease. Finally, other factors may be contributed to SFTSV infection and fatal outcome. For example, life style (tea peaking), high positive rate of ticks in their living areas and fatigue might also be the determinants for SFTSV infection. Furthermore, the death of case A could make a great affection on case B and case C, especially for B who shared a common tea leaves picking experience with A. After case A died, case C who was 79 years old with hypertension and heart diseases, traveled from Changxing to Linhai, Ningbo and Linhai in three days, which was a great challenge to her body and psychology. These factors might also be attributed to fatal outcome. Further research should be conducted identify other determinants for SFTSV infection and fatal outcome.

To the best of our knowledge, this is the first report about genetic susceptibility of SFTSV infection. Our findings mean that genetic susceptibility is one of determinants for severe fever with thrombocytopenia syndrome virus infection and fatal outcome. These indicated that once a person was infected with SFTSV, his family members had a greater chance to be infected with SFTSV. Further study should be done to study genetic predisposition of SFTSV infection.

### Ethical Approval

Experimental research reported in this study has been performed with the approval of the ethics committee of Zhejiang Provincial Centre for Disease Control and Prevention (Zhejiang CDC). Human research was carried out in compliance with the Helsinki Declaration. All participants provide their written informed consent to participate in this study.

## References

[1] Yu XJ, Liang MF, Zhang SY, Liu Y, Li JD, Sun YL, et al (2011) Fever with thrombocytopenia associated with a novel bunyavirus in China. N Engl J Med 364: 1523–1532. 10.1056/NEJMoa1010095 21410387 PMC3113718

[2] Kang K, Tang XY, Xu BL, You AG, Huang XY, Du YH, et al (2012) Analysis of the epidemic characteristics of fever and thrombocytopenia syndrome in Henan Province, 2007–2011. Chin J Prev Med 46: 106–109. 22490189

[3] Zhang YZ, He YW, Dai YA, Xiong YW, Zheng H, Zhou DJ, et al (2012) Hemorrhagic fever caused by a novel bunyavirus in China: pathogenesis and correlates of fatal outcome. Clin Infect Dis 54: 527–533. 10.1093/cid/cir804 22144540

[4] Liu W, Lu QB, Cui N, Li H, Wang LY, Liu K, et al (2013) Case-fatality ratio and effectiveness of Ribavirin therapy among hospitalized patients in China who had severe fever with thrombocytopenia syndrome. Clin Infect Dis 57: 1292–1299. 10.1093/cid/cit530 23965284

[5] Gai ZT, Zhang Y, Liang MF, Jin C, Zhang S, Zhu CB, et al (2012) Clinical progress and risk factors for death in severe fever with thrombocytopenia syndrome patients. J Infect Dis 206: 1095–1102. 10.1093/infdis/jis472 22850122

[6] Sun YL, Jin C, Zhan FX, Wang XJ, Liang MF, Zhang QF, et al (2012) Host cytokine storm is associated with disease severity of severe fever with thrombocytopenia syndrome. J Infect Dis 206: 1085–1094. 10.1093/infdis/jis452 22904342

[7] http://www.moh.gov.cn/mohwsyjbgs/s8348/201010/49272.shtml.

[8] Zhang L, Wang XY, Zhang YJ, Zhang YL, Wang HL, Wang CW, et al (2012) Severe fever with thrombocytopenia syndrome Bunyavirus (SFTSV) infections in Zhejiang Province, China. Inter J Infect Dis 17: e137–138. 10.1016/j.ijid.2012.07.007 23103388

[9] Jiao Y, Zeng XY, Guo XL, Qi X, Zhang X, Shi ZY, et al (2012) Preparation and evaluation of recombinant severe fever with thrombocytopenia syndrome virus nucleocapsid protein for detection of total antibodies in human and animal sera by double-antigen sandwich enzyme-linked immunosorbent assay. J Clin Microbiol 50: 372–377. 10.1128/JCM.01319-11 22135253 PMC3264160

[10] Bao CJ, Qi X, Wang H. (2011) A novel Bunyavirus in China. N Engl J Med 365: 862–863. 10.1056/NEJMc1106000#SA1 21879913

[11] Bao CJ, Guo XL, Qi X, Hu JL, Zhou MH, Varma JK, et al (2011) A family cluster of infections by a newly recognized Bunyavirus in eastern China, 2007: further evidence of person-to-person transmission. Clin Infect Dis 53: 1208–1214. 10.1093/cid/cir732 22028437

[12] Gai ZT, Liang MF, Zhang Y, Zhang S, Jin C, Wang SW, et al (2012) Person to person transmission of severe fever with thrombocytopenia syndrome Bunyavirus through blood contact. Clin Infect Dis 54: 249–252. 10.1093/cid/cir776 22095565 PMC3245727

[13] Liu Y, Li Q, Hu WF, Wu JB, Wang YB, Mei L, et al (2012) Person-to-person transmission of severe fever with thrombocytopenia syndrome virus. Vector-Borne Zoonot 12: 156–160. 10.1089/vbz.2011.0758 21955213

[14] Tang XY, Wu WL, Wang HF, Du YH, Liu LC, Kang K, et al (2013) Human-to-Human transmission of severe fever With thrombocytopenia syndrome Bunyavirus through contact with infectious blood. J Infect Dis 207: 736–739. 10.1093/infdis/jis748 23225899

[15] Chen HB, Hu K, Zou JJ, Xiao JX. (2013) A cluster of cases of human-to-human transmission caused by severe fever with thrombocytopenia syndrome Bunyavirus. Inter J Infect Dis 17: e206–e208. 10.1016/j.ijid.2012.11.006 23218674

[16] Sun JM, Chai CL, Lv HK, Lin JF, Wang CW, Chen EF, et al (2014) Epidemiological characteristics of severe fever with thrombocytopenia syndrome in Zhejiang Province, China. Inter J Infect Dis 25: 180–185. 10.1016/j.ijid.2014.02.022 24947422

[17] Sun JM, Zhang YJ, Gong ZY, Zhang L, Lv HK, Lin JF, et al (2015) Seroprevalence of severe fever with thrombocytopenia syndrome virus in southeastern China and analysis of risk factors. Epidemiol Infect 143: 851–856. 10.1017/S0950268814001319 24866248 PMC4411641

[18] Wang ML, Lai JH, Zhu Y, Zhang HB, Li C, Wang JP, et al (2009) Genetic susceptibility to haemorrhagic fever with renal syndrome caused by Hantaan virus in Chinese Han population. Inter J Immunogenet 36: 227–229. 10.1111/j.1744-313X.2009.00848.x 19473214

[19] Coffey LL, Mertens E, Brehin AC, Fernandez-Garcia MD, Amara A, Despres P, et al (2009) Human genetic determinants of dengue virus susceptibility. Microbes Infect 11: 143–156. 10.1016/j.micinf.2008.12.006 19121645

[20] Loeb M, Eskandarian S, Rupp M, Fishman N, Gasink L, Patterson J, et al (2011) Genetic Variants and Susceptibility to Neurological Complications Following West Nile Virus Infection. J Infect Dis 204:1031–1037. 10.1093/infdis/jir493 21881118 PMC3203390

[21] Abdel-Ghafar AN, Chotpitayasunondh T, Gao Z, Hayden FG, Nguyen DH, de Jong MD, et al (2008) Update on avian influenza A (H5N1) virus infection in humans. N Engl J Med 358: 261–273. 10.1056/NEJMra0707279 18199865

[22] Horby P, Nguyen NY, Dunstan SJ, Baillie JK. (2012) The role of host genetics in susceptibility to influenza: a systematic review. PLoS One 7: e33180 10.1371/journal.pone.0033180 22438897 PMC3305291

[23] Liu YX, Li SY, Zhang GL, Nie G, Meng ZZ, Mao DT, et al (2013) Genetic variants in IL1A and IL1B contribute to the susceptibility to 2009 pandemic H1N1 influenza A virus. BMC Immunol 14: 37 10.1186/1471-2172-14-37 23927441 PMC3750637

